# Totally extraperitoneal repair of inguinal hernia: Sir Ganga Ram Hospital technique

**DOI:** 10.4103/0972-9941.27731

**Published:** 2006-09

**Authors:** Pradeep K Chowbey, Rajesh Khullar, Anil Sharma, Vandana Soni, Manish Baijal

**Affiliations:** Minimal Access and Bariatric Surgery Centre, Sir Ganga Ram Hospital, New Delhi - 110 060, India

**Keywords:** Totally extraperitoneal repair, SGRH technique, finger glove balloon, rolled mesh

## Abstract

Laparoscopic approach for hernia has evolved rapidly over the past decade. We adopted the TEP repair early as we believe in preserving the sanctity of the coelomic cavity. Once well versed with the approach we have found it an efficient and cost effective method for groin hernia repair.

Endoscopic totally extraperitoneal hernia repair is a technically demanding procedure. Indepth anatomical knowledge, training and advanced technical skill is needed for the surgeon to perform this procedure. To make the procedure cost effective and prevent hernia recurrences, we have modified and innovated to simplify the procedure.

This modification which we have named the SGRH technique, innovates by creating the preperitoneal working space with the help of an indigenous glove finger balloon. A rolled mesh makes placement and fixation easier in the limited working space. The mesh is unrolled on the peritoneal surface (floor), a manouver which is technically simpler. On desufflation the mesh comes to appose the Fruchad's orifice covering all potential hernial sites. With the modified SGRH technique we have found TEP to be safe, cost effective, reproducible and without significant complications.

The inguinal hernia repair has been a controversial area in the surgical practice from the time it has been conceived. The history of inguinal hernia repair over several decades implies how innovations are adopted into surgical practice through a combination of scientific and subjective processes. The techniques of laparoscopic hernia repair have evolved in parallel with experience and technology. In the laparoscopic procedure, tension-free repair is achieved by placement of a prosthetic mesh to cover the entire groin area, including the sites of direct, indirect and femoral hernia. The laparoscopic approach is based on the principle of tension-free repair, which has been well established by open operation of Nyhus and Stoppa. The greater availability of space in the extraperitoneal approach facilitates the insertion of a much bigger mesh.

## Patient selection

Totally extraperitoneal (TEP) groin hernia repair is an advanced laparoscopic procedure. It requires greater skills of laparoscopic dissection and manipulation as the working space available is limited. It has a long learning curve and must be done only after acquiring experience in basic laparoscopic procedures and when the learning curve is over. Today, we are well past the learning curve and have performed well over thousand laparoscopic groin hernia repairs. Except for strangulated hernia, at present there are no absolute contraindications for this procedure. Relative contraindications include patients unfit for anesthesia, obese and pregnant patients and patients with a history of lower abdominal surgery.

## Preoperative preparation

A thorough history of the presenting complaints and other comorbid conditions should be taken. Specific measures should be taken if the patient is on drugs like ASA and warfarin oral hypoglycemic agents, etc. Besides routine hematological investigations, other specific investigations like X-ray chest, ECG, coagulation profile, pulmonary function test, etc, should be done for patients with history of cardiac / pulmonary pathology.

A written consent should be obtained after explaining the probable complications and possibility of conversion to open surgery.

Following pre-anesthetic check-up and clearance for surgery, the patient is kept fasting overnight. The patient is prepared adequately.

## Surgical technique

The procedure is done under general anesthesia (regional anesthesia if the patient is unfit for general anesthesia). The patient is catheterized and prophylactic antibiotic is given at the time of induction of anesthesia. After induction, complete reduction of the contents of the hernial sac is ensured.

## Extraperitoneal access

**Figure 1:**
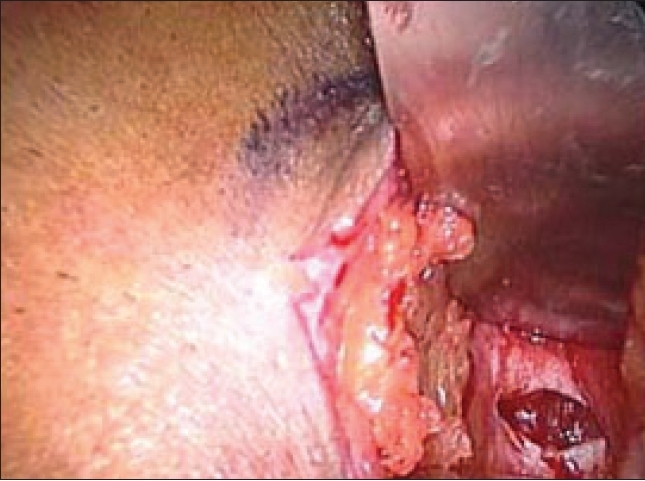
Transverse infraumbilical incision, with incision in anterior rectus sheath

**Figure 2:**
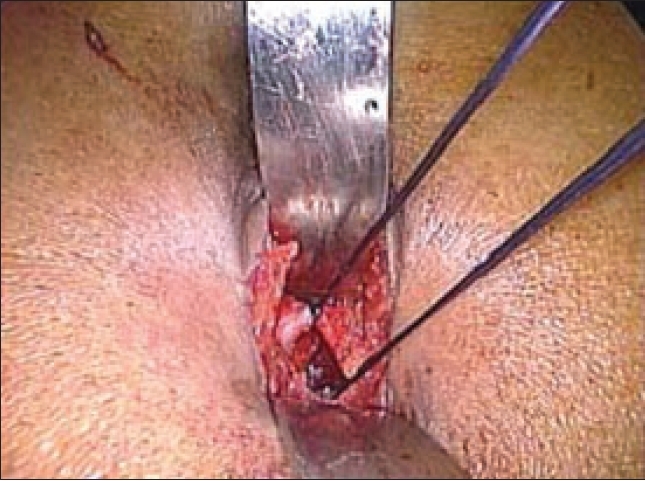
Stay sutures over incised anterior rectus sheath

A 10-mm infraumbilical transverse incision is made. The anterior rectus sheath is exposed and transverse incision is then made on the anterior rectus sheath to one side of the midline to avoid inadvertent opening of the peritoneum [Figure 1]. The margins of incised sheath are held in stay sutures using vicryl 1–0 [Figure 2]. The rectus muscle is retracted laterally from the midline and by finger dissection a space is created between the rectus muscle and the posterior rectus sheath.

## Balloon dissection of the extraperitoneal space

**Figure 3:**
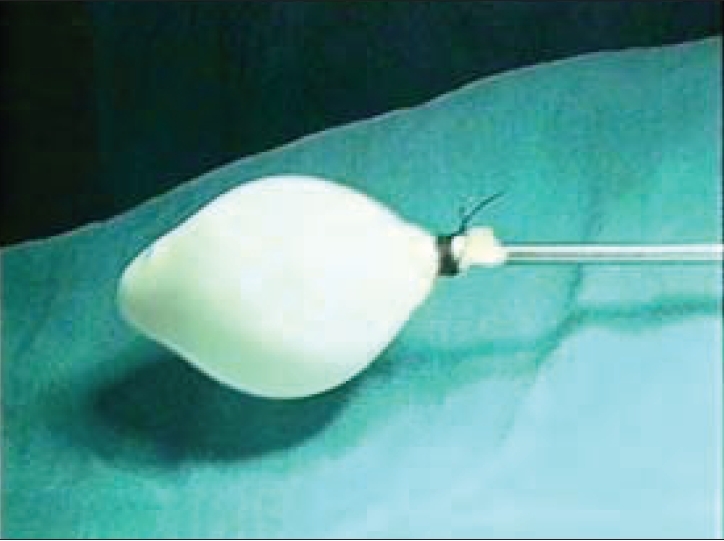
Indigenous balloon trocar

A self-made balloon is then inserted in the preperitoneal space created. The balloon trocar used by us is an indigenously made trocar, where we tie two finger stalls of size 8 latex surgical glove on the tip of the 5-mm laparoscopic suction cannula [Figure 3]. The balloon trocar is then inflated with 100–150 ml of saline. It not only creates an initial working space but also brings about hemostasis by balloon tamponade. The balloon is then deflated and the cannula is removed.

**Figure 4:**
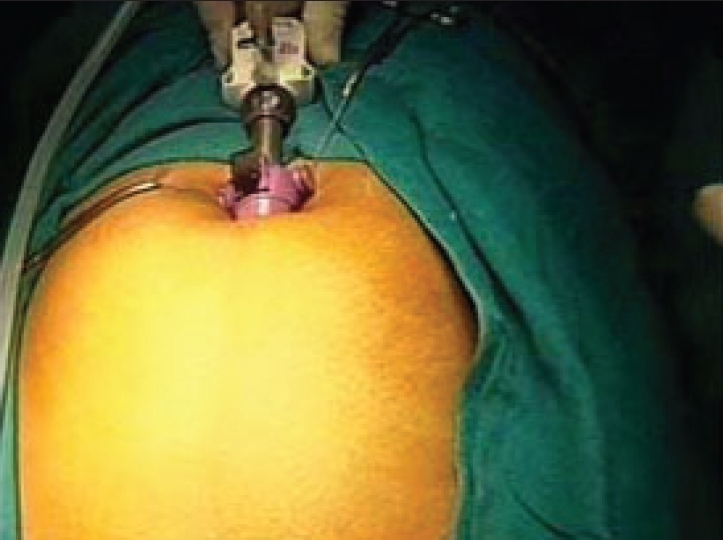
Hasson's cannula introduced in subumbilical port

A 10-mm Hasson's Cannula (blunt tip cannula) is then introduced into the preperitoneal space through the infraumbilical incision [Figure 4]. The Hassan's cannula snuggly fits into the incision and is secured with stay sutures. The insufflation tubing is attached to the Hasson's Cannula and insufflation is begun with pressure setting at 12 mmHg.

**Figure 5:**
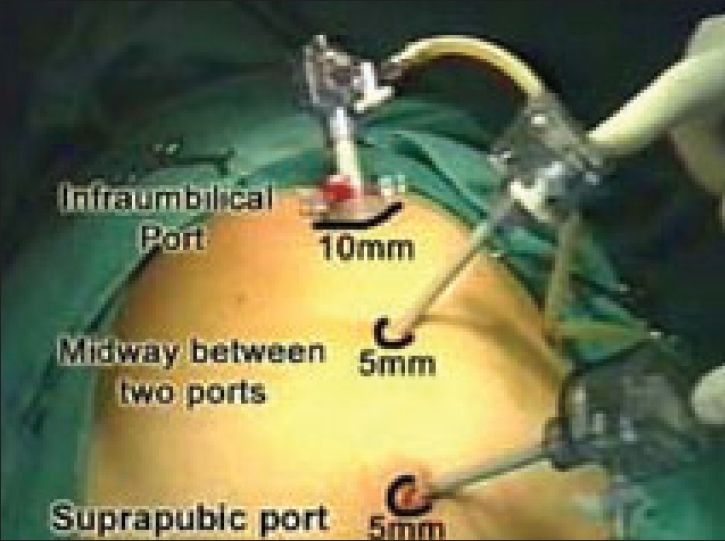
Port placement for totally extraperitoneal

**Figure 6:**
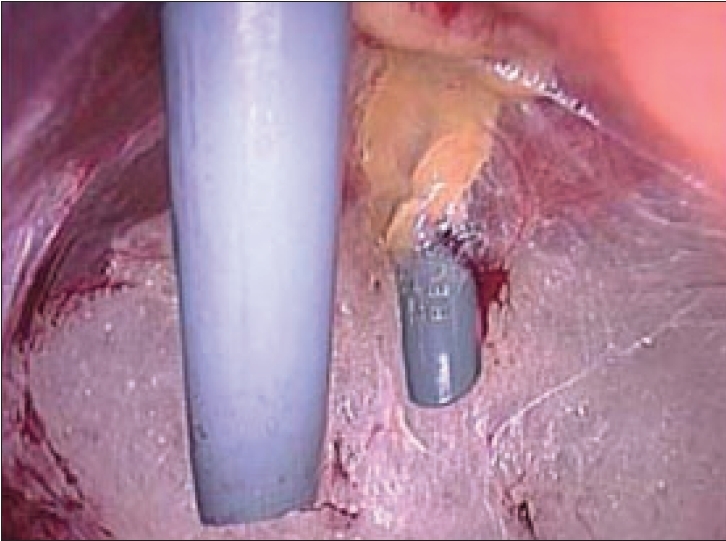
Preperitoneal space

A 10-mm 30° telescope is used. The camera is introduced through the subumbilical port and preperitoneal space is visualized. The other two working ports are placed in the preperitoneal space. First, a 5-mm port is placed about 2–3 cm above the pubic symphysis in the midline; and second, a 5/10 mm port is placed in the midline, midway between the two placed ports (subumbilical and suprapubic) [Figures 5 and 6].

## Dissection of the extraperitoneal space

**Figure 7:**
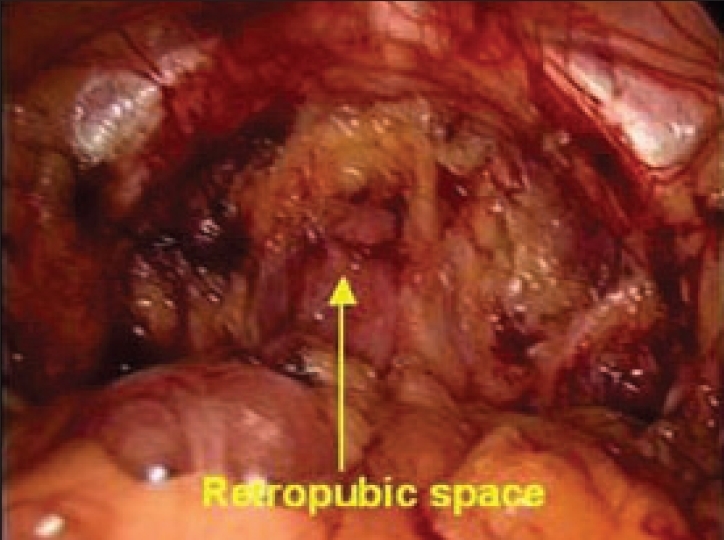
Retropubic space after dissection

The surgeon stands on the side opposite the operating side or side where hernia is present. Dissections in extraperitoneal space are begun by dividing the loose aerolar tissue in the midline using sharp and blunt dissection. The first landmark / reference point, i.e., the pubic bone, is identified - which appears as a white glistening structure in the midline. The pubic bone is visualized and bared of all connective tissue, creating a shelf extending about 2–3 cm in the retropubic space, which acts as a shelf to place the mesh [Figure 7].

**Figure 8:**
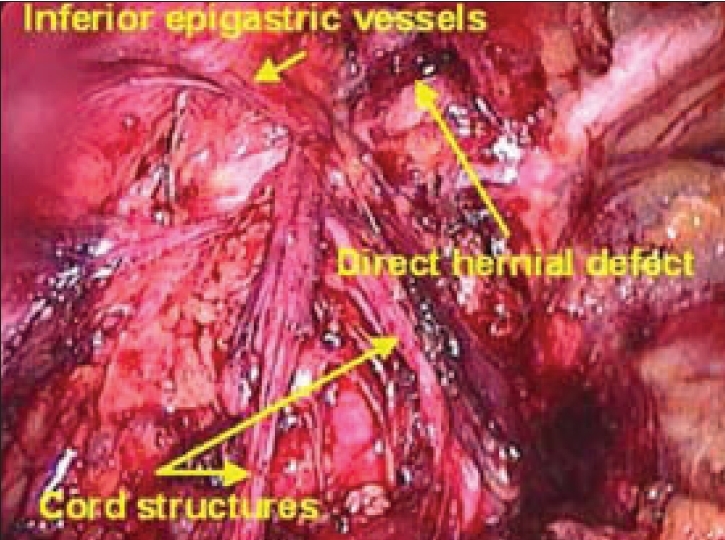
Left direct hernial defect seen after dissection

**Figure 9:**
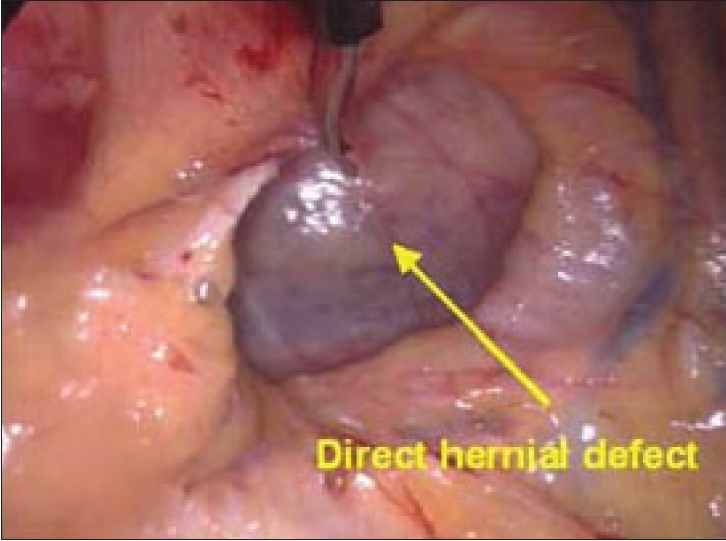
Right direct hernial defect

The dissection is then traced laterally towards the side of the hernia. In case of direct hernia, the hernial sac is visualized going into the weakness in the Hasselbach's triangle before the inferior epigastric vessels can be visualized. On the other hand, in the indirect hernia, the inferior epigastric vessels are seen before the hernial sac is encountered. Once the adhesions are lysed or hernial sac is reduced, as in direct hernia, the anatomical landmarks which now become visible are Cooper's ligament, iliopubic tract, femoral canal and the inferior epigastric vessels [Figures 8, 9].

**Figure 10:**
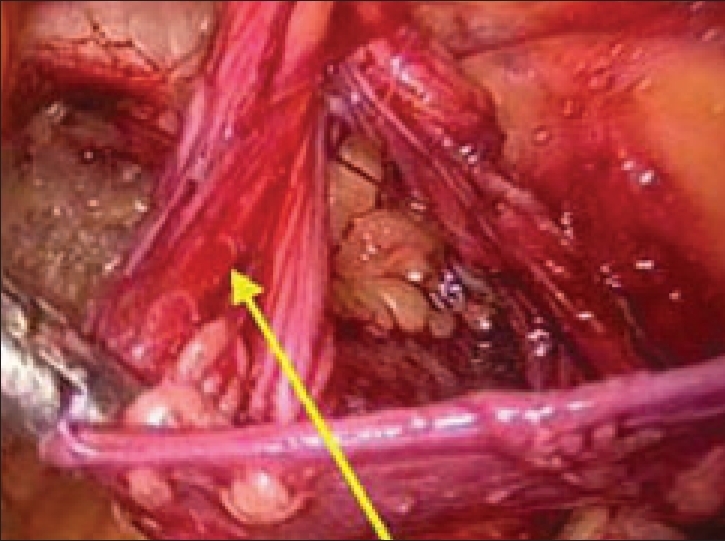
Right indirect hernial sac with cord structures

**Figure 11:**
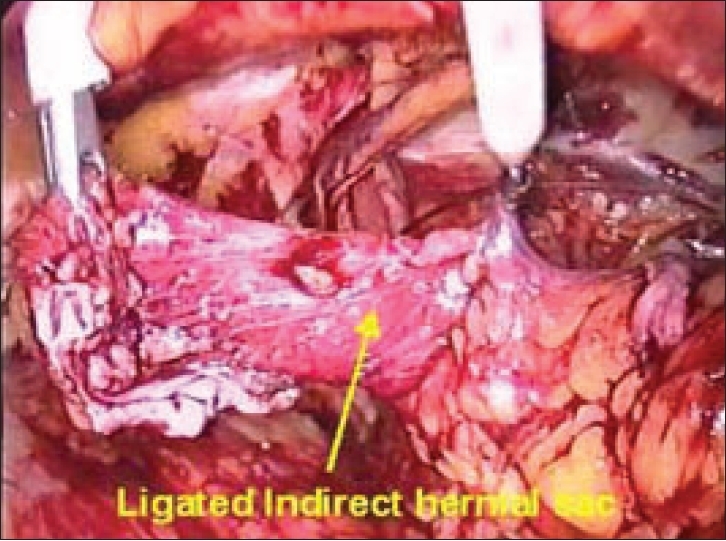
Dissected and ligated right indirect hernial sac

The spermatic cord lies immediately inferior and lateral to the inferior epigastric vessels. The adhesions all around the cord are lysed with caution as the external iliac vessels lie just below the cord structures. The peritoneal extension (sac) is seen as a white glistening structure lying anterolateral to the cord [Figure 10]. The sac is completely dissected off the cord structures and reduced. In cases of complete hernia, attempt should not be made to completely reduce the sac as excessive traction and dissection cause severe postoperative pain and edema. The sac should be transected and ligated using a catgut endoloop or by intracorporeal sutures, leaving the distal sac open *in situ* [Figure 11].

The peritoneal sac with reflection is completely reduced. The vas deferens is seen lying separately on the medial side and gonadal vessels are seen on the lateral side forming a triangle. This triangle, known as ‘triangle of doom,’ is bound medially by the vas deferens, laterally by gonadal vessels with its apex at the internal inguinal ring and the basic is formed by the peritoneum. No dissection should be carried within this triangle as it contains the external iliac vessels.

Dissection is continued lateral to the cord structures to create adequate space for the placement of mesh. The lateral space contains loose aerolar tissue, which is completely divided using sharp and blunt dissection. The psoas muscle is seen lying on the floor on which lateral cutaneous nerve of thigh and genitofemoral nerve can be seen traversing. The lateral boundary of the dissection is marked by the anterior superior iliac spine.

## Placement of mesh

**Figure 12:**
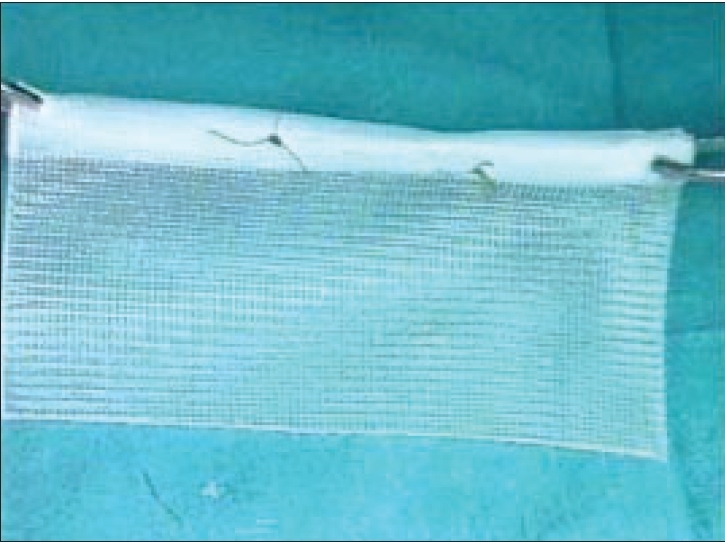
Rolled prolene mesh

**Figure 13:**
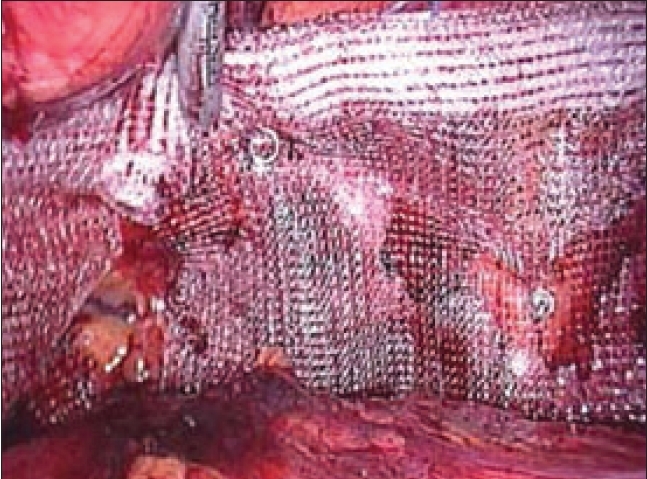
Prolene mesh being placed for repair

After creating the lateral space adequately, the mesh is introduced through the 10-mm subumbilical port [Figure 12]. The mesh is placed over the space created so that it covers the sites of direct, indirect, femoral and obturator hernias [Figure 13]. The mesh is then secured in place with the help of fixation devices like helical fasteners, staples, anchors, etc, depending upon the preference of the surgeon. After adequately spreading the mesh, which extends from the midline medially, to lying over the psoas muscle on the lateral side, preperitoneal space is deflated.

In cases of bilateral hernias, a similar procedure can be done on both the sides through the same three ports made for unilateral repair.

